# Biochar from commercially cultivated seaweed for soil amelioration

**DOI:** 10.1038/srep09665

**Published:** 2015-04-09

**Authors:** David A. Roberts, Nicholas A. Paul, Symon A. Dworjanyn, Michael I. Bird, Rocky de Nys

**Affiliations:** 1 MACRO – the Centre for Macroalgal Resources and Biotechnology, College of Marine and Environmental Sciences, James Cook University, Townsville Australia 4811; 2 National Marine Science Centre, Southern Cross University, Coffs Harbour Australia 2450; 3 Centre for Tropical Environmental and Sustainability Sciences, College of Science, Technology and Engineering, James Cook University, Cairns Australia 4870

## Abstract

Seaweed cultivation is a high growth industry that is primarily targeted at human food and hydrocolloid markets. However, seaweed biomass also offers a feedstock for the production of nutrient-rich biochar for soil amelioration. We provide the first data of biochar yield and characteristics from intensively cultivated seaweeds (*Saccharina*, *Undaria* and *Sargassum* – brown seaweeds*,* and *Gracilaria, Kappaphycus* and *Eucheuma* – red seaweeds). While there is some variability in biochar properties as a function of the origin of seaweed, there are several defining and consistent characteristics of seaweed biochar, in particular a relatively low C content and surface area but high yield, essential trace elements (N, P and K) and exchangeable cations (particularly K). The pH of seaweed biochar ranges from neutral (7) to alkaline (11), allowing for broad-spectrum applications in diverse soil types. We find that seaweed biochar is a unique material for soil amelioration that is consistently different to biochar derived from ligno-cellulosic feedstock. Blending of seaweed and ligno-cellulosic biochar could provide a soil ameliorant that combines a high fixed C content with a mineral-rich substrate to enhance crop productivity.

Seaweed (marine macroalgae) aquaculture is a rapidly expanding industry with global annual production now exceeding 19 million tonnes (Mt) landed weight^1^. The majority of seaweed is cultivated for the production of food and hydrocolloids, however it also represents a possible feedstock for the production of biochar – a C-rich “biological charcoal”^2^. Biochar is produced through the process of slow pyrolysis in which biomass is combusted under oxygen limitation. Biochar has a recalcitrant C content and therefore offers a form of long-term carbon (C) sequestration, particularly when biochar is amended to agricultural soils as a source of soil C. When applied to soils, biochar can also improve soil fertility by increasing the retention of nutrients and reducing the emissions of N_2_O from agricultural soils^3^,^4^. Consequently, biochar has been widely proposed as a means of delivering soil C sequestration that also assists in the remediation of degraded and low-fertility soils^3^. In addition, biochar also has emerging applications as a source of bioenergy^5^ and in the treatment of contaminated waste water^6^.

The majority of biochar that is produced is derived from terrestrial ligno-cellulosic feedstocks (“woody” plants) that yield biochar with a high fixed C content (typically in excess of 70%) but low direct nutritive value. In contrast, it has recently been demonstrated that biochar can be produced from seaweed to yield a product with several defining characteristics for applications in diverse fields^2^,^3^. Seaweed often yields high proportions of biochar in comparison to ligno-cellulosic feedstock and, while generally lower in C content than ligno-cellulosic biochar, seaweed biochar has a high exchangeable nutrient content and Cation Exchange Capacity (CEC)^2^. The high CEC of seaweed biochar makes it a highly effective in the retention of nutrients in agricultural soils. It also makes it an effective biosorbent of metals from waste water effluents, and seaweed biochar can then be modified to increase its affinity for problematic oxyanionic contaminants such as selenium (Se) that otherwise show limited affinity for passive biosorbents^7^,^8^,^9^. Despite the diverse applications of seaweed biochar, there have been few studies that consider the production of biochar from a range of seaweed feedstocks. Studies that have been conducted on seaweed biochar have focused on seaweeds from non-commercial sources and bioremediation processes^2^. However, for the large-scale production of seaweed biochar to become feasible, it will be necessary to convert seaweeds cultivated at commercial scales.

Here we provide the first data on the yield and characteristics of biochar produced from species of seaweed that are cultivated at scale and which are commercially available. This study has two main aims. First, we produce biochar from commercially available seaweed feedstock that represents the predominant biomass sources currently available for large-scale biochar production (~15 million t yr^−1^, ~78% global annual seaweed production^1^). Second, we contrast the properties of seaweed biochar as a function of geographical origin, species and division (“red” vs “brown” seaweeds) to determine whether seaweed biochar is consistent across these factors. To do this, we produce biochar from *Gracilaria edulis*, *Eucheuma spinosum* and *Kappaphycus alvarezii* (red seaweed produced for the extraction of the hydrocolloids agar and carrageenan, ~7.25 million t yr^−1^), and *Saccharina japonica*, *Undaria pinnatifida* and *Sargassum* sp. (brown seaweed produced for food – “kombu” or “wakame” – and the hydrocolloid alginate, ~7.75 million t yr^−1^) (hereafter referred to by genus). Collectively these species account for 75% of global seaweed cultivation and are immediately available to support large-scale environmental biotechnologies based on biochar.

## Results

### Biochar production and characterisation

Seaweed biochar was produced from six species of seaweed and each of these species was collected from two geographically distinct locations that are representative of the dominant sources of each biomass feedstock (Table 1). Three independent biochar samples were produced from each species collected at each location to account for any heterogeneity in the sample and in the pyrolysis process itself. Three of the species were red seaweeds (*Gracilaria*, *Eucheuma* and *Kappaphycus*) and three were brown seaweeds (*Saccharina*, *Undaria* and *Sargassum*).

The six species of seaweed yielded between 45-62% biochar after slow pyrolysis for 60 14min at 450°C and biochar yield did not differ significantly between biochar produced from red and brown species of seaweed (t < 2.776, P = 0.375, df = 4). The biochar samples were relatively consistent with respect to their C, H and O contents regardless of species, location or division (ranging from 22–35%, 1.1–2.8% and 14–25% for C, H and O respectively) and the organic C content comprised more than 85% of the total C content for all samples (Table 1).

A clear characteristic of all seaweed biochars was the high concentrations of N, P and K. N ranged from 0.3–2.8%, P from 0.5–6.6 g kg^−1^, and K from 5.1–119 14g kg^−1^ (Table 1). Biochar produced from species of brown seaweed had, on average, a slightly higher C and H content and lower S content than biochar produced from species of red seaweeds. The energetic value of the seaweed biochars, as estimated by the Higher Heating Value (HHV), ranged from 10.7–17.8 14MJ kg^−1^ (Table 1). The C/N ratio of the seaweed biochars ranged from 11–74 in the samples (Table 1). All seaweed biochar samples had an alkaline pH, ranging from 7.6 to 11.2 (Table 2). The biochar produced from brown seaweed had a higher pH (>9.9) than biochar from red seaweed (<9.0) (Table 2). Biochar produced from *Eucheuma* had a higher surface area (30 14m^2^ g^−1^) than biochar produced from the other species (<10 14m^2^ g^−1^) (Table 2). The seaweed biochar samples had negligible or no exchangeable Al, but high levels of the remaining exchangeable cations (Ca, K, Mg and Na) (Table 3).

### Variability in biochar properties between locations, species and divisions

**Figure 1:**
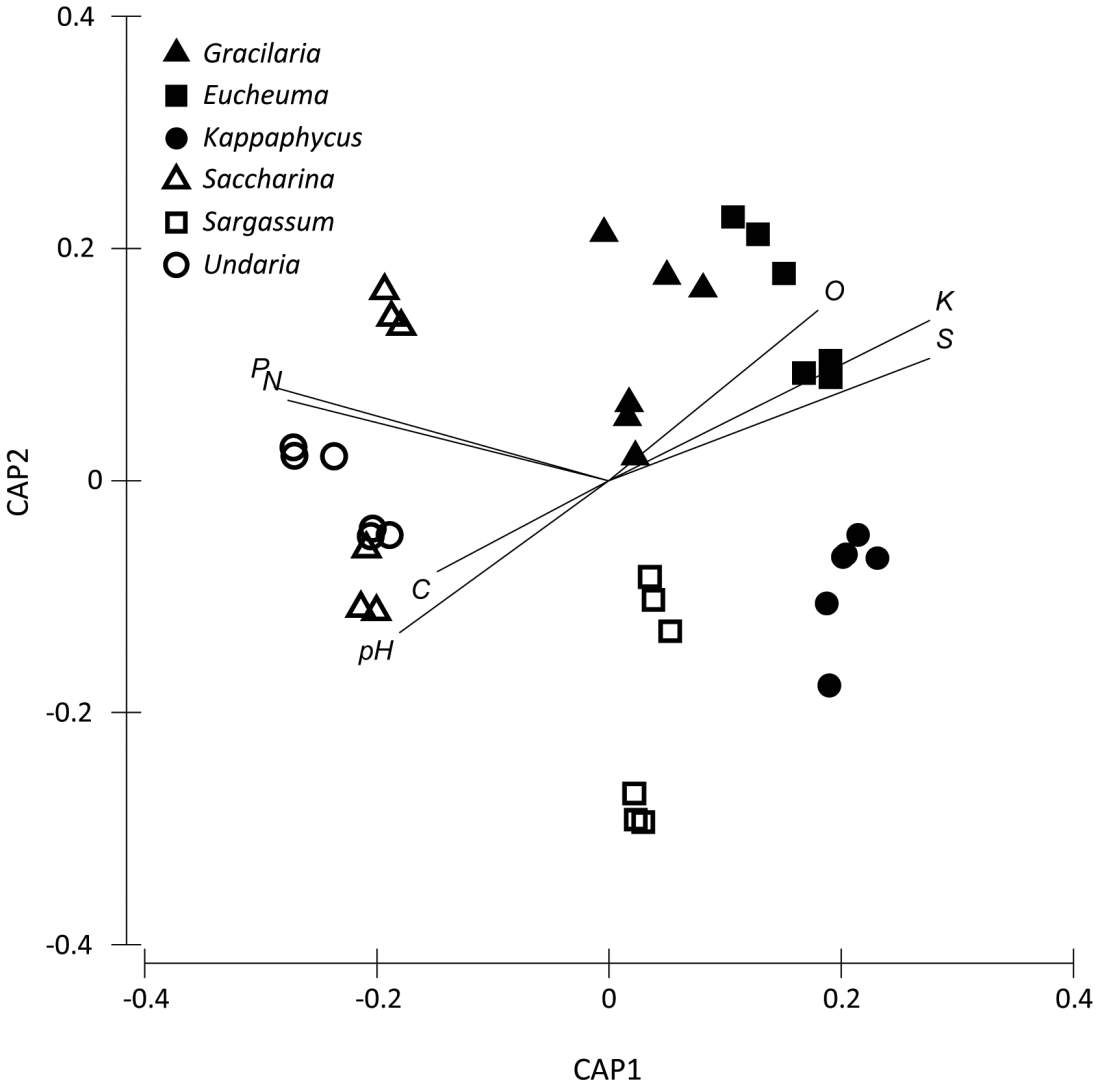
Canonical Analysis of Principal Coordinates (CAP) ordination of seaweed biochar characteristics with vector overlay of Spearman rank correlations of individual biochar properties. The vector overlay is limited to variables having lengths of at least 0.5. The variables included in the analysis include biochar yield, elemental profile (C, H, O, N, S, P and K) and pH. Black symbols show species of red seaweeds, white symbols show species of brown seaweeds. The distinct sample origins for each species are visible by the clustering of the three replicates for each “species x origin” combination.

The biochars produced from species of red and brown seaweeds had different elemental compositions, with relatively clear clustering of red and brown seaweed biochars in the CAP ordination. This ordination included the elemental profiles of the biochars (C, H, O, N, S, P and K), as well as the yield and pH as input variables (Figure 1). Biochars produced from red seaweeds had higher concentrations of S and K and lower concentrations of C and H than the biochars produced from brown seaweeds (C: t = 3.518, P = 0.025; H: t = 4.023, P = 0.016; S: t = 3.570, P = 0.023 and K: t = 4.871, P = 0.008, df = 4). The biochars produced from brown seaweeds had a higher pH than the biochars produced from red seaweeds (pH: t = 5.902, P = 0.004), and tended to have a higher P and N content, although these differences were not statistically significant (Figure 1, Tables 1 and 2). There was, however, some variation in most of the physico-chemical properties of the biochar between species collected from different locations. In particular, *Saccharina* from the two locations had very different concentrations of K (8.9 and 51.9 14g kg^−1^), while *Sargassum* from different locations had very different yield (49 and 62%) and S content (0.9 and 2.8%) (Table 1).

There were very large differences in the CEC of the seaweed biochars, particularly between samples of the same species collected from different locations. This was most evident for exchangeable K in *Undaria* and *Kappaphycus* which differed by an order of magnitude depending upon location (Table 3). All samples were characterized by no detectable exchangeable Al and high concentrations of exchangeable Ca, Mg and Na (Table 3) and there were no differences between biochar produced from red and brown species or seaweed with respect to exchangeable cations (t < 2.50, P > 0.05 for all exchangeable cations).

## Discussion

While some biochar properties varied between species and location, seaweed as a feedstock was relatively consistent in the sense that all species yielded high amounts of biochar per unit biomass, and the resulting biochars were relatively low in C, but rich in nutrients (N, P, K, Ca and Mg) and with a basic pH. There was, unsurprisingly, some variability in the characteristics of seaweed biochar produced from species collected from different locations. Further research should clearly consider environmental factors that might affect the spatial and temporal variability in seaweed biochar properties if large-scale production from cultivated seaweed is to occur. Nevertheless, the overriding conclusion of our work is that seaweed biochar is fundamentally different to biochar produced from ligno-cellulosic feedstock. Seaweed yields more biochar than ligno-cellulosic biomass during pyrolysis, and the resulting biochar has a relatively low C content, but high concentrations of N, P and exchangeable K and Na relative to ligno-cellulosic feedstock. The seaweed biochars also have a much lower BET surface area than ligno-cellulosic biochar^10^. Seaweed biochar is consistently different to ligno-cellulosic biochar with respect to each of these properties, regardless of species or location and these findings are consistent with previous results for biochar from non-commercial macroalgae^2^ and microalgae^11^.

The combination of the low C content but high mineral content of seaweed biochar results in a low HHV of seaweed biochar in comparison to ligno-cellulosic biochars, which can exceed 30 14MJ kg^−1^^12^,^13^. While the elemental profile of seaweed biochar limits its HHV, it also makes seaweed biochar a unique substrate that may be tailored to agricultural applications^3^. Seaweed biochars have a relatively consistent elemental composition that was more similar to biochar from manure than from ligno-cellulosic feedstocks^2^ and the low C/N ratio is particularly important in this context. The C/N ratio estimates the ability of organic substrates in the biochar to mineralize and release inorganic N when applied to soils. Typically, a C/N > 20–30 suggests N will not be available to plants^14^. As ligno-cellulosic biochars have high C and low N contents (and consequently high C/N ratios), the benefits of ligno-cellulosic biochar application to soils is indirect through improved nutrient retention^14^. In contrast, *Gracilaria*, *Saccharina* and *Undaria* biochars have a C/N ratio < 20, indicating they could directly contribute bioavailable N and P to soils, in addition to enhancing the retention of supplemental nutrients provided in the form of fertilizer. This prediction is supported by the known beneficial effects of other seaweed biochars on crop production^3^.

The main limitation of seaweed biochars that requires consideration is the high concentration of exchangeable Na which could increase soil salinity. Previous research suggests that the Na component of seaweed biochar is leachable, but levels are within biosolids limits, and overall positive short-term effects on crop productivity have been described following the application of seaweed biochar to low fertility soils^3^. Nevertheless, it may be necessary to apply the biochar to soils well in advance of cropping to allow exchangeable Na to be leached from the biochar^15^. An additional approach to the production of biochar from seaweed biomass could be to blend it with ligno-cellulosic biomass to dilute the Na content of the resulting biochar. Indeed, the targeted blending of seaweed and ligno-cellulosic biochar may be a particularly strategic approach as it would yield a biochar that combines the nutrient- and mineral-rich properties of seaweed biochar with C-rich ligno-cellulosic feedstock. This blended biochar could be more suited to delivering stable C accrual in agricultural soils. For example, under the Carbon Farming Initiative (CFI) in Australia land owners are given financial incentives to adopt land management practices that result in soil C accrual^16^. However, when one factors in the cost of N addition that is required to stabilize C accrual, the costs of achieving C accrual typically outweigh any profits that may be realized through C credits^16^. The production of N- and C-rich blended biochar from seaweed and ligno-cellulosic feedstock could yield a C-rich soil ameliorant that also includes a significant component of exchangeable N to stabilize soil C accrual.

Increased seaweed cultivation has been proposed as a sink of “Blue Carbon” in climate change mitigation strategies^17^,^18^. If one assumes that the 19 14Mt wet (landed) weight of cultivated seaweed equates to 1.9 14Mt dry weight (a 10:1 wet to dry ratio)^18^, and a biochar yield of 59% with a mean C_Org_ content of 30%, the scope for C sequestration from seaweed biochar derived from commercially cultivated seaweeds is 0.33 14Mt C yr^−1^. These figures do not take into account energy costs in the production process as the energy balance of biochar production varies greatly with the scale of the production system^19^. Regardless, the C sequestration potential for biochar produced from cultivated seaweeds is small relative to anthropogenic C emissions. However, seaweed biochar could deliver significant improvements in soil C accrual indirectly as a soil ameliorant to enhance crop production. Soil accounts for 20% of the global capture of anthropogenic CO_2_ emissions each year, but is also a non-renewable resource that is being increasingly degraded^20^. The unique properties of seaweed biochar provide an opportunity for it to be blended with ligno-cellulosic biochar to produce targeted products for broad-spectrum agricultural applications. Blending of mineral-rich seaweed biochars with C-rich ligno-cellulosic biochar could yield unique soil ameliorants that are specifically created to match the requirements of specific types of soil^21^.

In conclusion, we found that biochar can be produced from a range of commercially cultivated seaweeds to yield unique ameliorants that could be applied to improve soil fertility. Biochar produced from red species of seaweed has higher concentrations of K and S, and lower C, H and pH than biochar produced from brown species of seaweed. However, while some properties of seaweed biochar unsurprisingly vary between seaweed divisions and origin of seaweed feedstock, seaweed biochar is consistently different to biochar produced from ligno-cellulosic feedstock with respect to its key characteristics, having low C content but high concentrations of exchangeable nutrients (particularly N, P, K, Ca and Mg). Therefore, opportunistic harvesting of seaweed blooms such as those that regularly occur in China^22^ and France^23^, as well as the production of seaweed in bioremediation processes^24^,^25^ could be appropriate and sustainable feedstock to support expansions in the production of seaweed biochar, in addition to the species of seaweed that are currently cultivated at large scales world-wide. Targeted blending of seaweed and ligno-cellulosic biochar could produce “designer” biochars that could be matched to specific soil types for broad-spectrum agricultural applications.

## Methods

### Biochar production

Six species of seaweed were obtained from commercial aquaculture suppliers and each species was collected from two geographically distinct regions. The six species of seaweed were *Gracilaria edulis*, *Eucheuma spinosum* and *Kappaphycus alvarezii* (Rhodophyta, “red seaweeds”), and *Sargassum* sp., *Undaria pinnatifida* and *Saccharina japonica* (Phaeophyceae, “brown seaweeds”) (hereafter referred to by genus). Each species was sampled from two geographically distinct regions (listed in Table 1) and the sample from each location was processed into biochar through slow pyrolysis three times independently to account for any heterogeneity in the sample and pyrolysis process. The seaweed samples were taken from homogenized biomass stockpiles at the wholesalers, who could provide a sample origin (region) but not a specific period of cultivation. For this reason, our comparisons and analyses of seaweed biochar in this study focus on differences between biochar produced from “red” and “brown” species of seaweed but no direct comparison of potential temporal variability in biochar properties. This analysis requires manipulative field experiments that are beyond the scope of this exploratory manuscript.

Each of the seaweed samples were converted to biochar independently using optimized production conditions that have been previously described^2^,^3^. First, a 200 14gram (g) sample of biomass from each species was rinsed in deionized water (DI) to remove surface salts and oven dried at 60°C to a constant mass (24 14h). Each sample was then milled in a stainless steel mill and sieved to retain the 1–2 14mm particle size fraction. The milled and sieved samples were then converted to biochar by slow pyrolysis. First, the dried seaweed was weighed to the nearest g and then placed into a ceramic mesh fiber basket. The basket was suspended inside a 2 14L stainless steel vessel (see schematic in Supplementary Figure S1). The vessel had a gas inlet that was connected to a high purity grade BOC N_2_ gas cylinder. The vessel was purged with N_2_ gas at a flow rate of 4.0 14L min^−1^ for 5 14min to vent air in the vessel. The purged vessel was then placed inside a ceramic-lined muffle furnace and was continuously purged with the N_2_ gas flowing at 4.0 14L min^−1^ while being heated to 450°C. The temperature inside the vessel was continuously monitored via a thermocouple inserted direct into the middle of the sample. Once the final hold temperature (450°C) was achieved, the samples were left in the furnace at a constant temperature for 60 14min After 60 14min the stainless steel vessel was removed from the furnace and the vessel and biochar was cooled to room temperature while N_2_ purging continued. The mass of biomass and resulting biochar was recorded to the nearest 0.1 14g before and after pyrolysis respectively to ascertain the yield of biochar, which was expressed as the % of original biomass retained as biochar.

### Biochar characterisation

Each biochar sample was characterized for a range of physico-chemical properties. The carbon (C), hydrogen (H), oxygen (O), nitrogen (N) and sulphur (S) contents of each sample were quantified in triplicate using an elemental analyser (OEA Laboratory Ltd, United Kingdom), while phosphorus (P and K) were measured by Inductively Coupled Plasma Optical Emissions Spectrometer (ICP-OES) after acid digest. First, 100 14mg of the biochar was placed in a Teflon digestion vessel with 3.0 14ml double distilled HNO_3_ and 1.0 14ml analytical grade H_2_O_2_. The solution was left for 2 14h at room temperature then heated in a microwave to 180°C for 10 14min, then finally diluted with Milli-Q water. The concentrations of P and K were measured with a Varian Liberty series II ICP-OES. Multi-element standard solutions containing the elements of interest were used for calibration and the results were reported after subtracting the procedural blanks. The standards were obtained from Choice Analytical (Sydney, Australia). The Higher Heating Value (HHV) was estimated on the basis of the elemental composition^26^. The pH of each biochar was determined in 10:1 water:CalCl_2_ mixtures according to Australian standard methods for soil analysis^27^. Brunauer, Emmet and Teller (BET) surface area was determined by N_2_ adsorption (Particle and Surface Sciences Pty Ltd., Gosford, New South Wales, Australia) and Cation Exchange Capacity (CEC) by silver thiourea (AgTU) extracts (Wollongbar Primary Industries Institute, Depart of Primary Industries, Wollongbar, New South Wales, Australia). Each of these analyses was conducted on a single sample of each species due to the large sample requirements for each measurement.

### Variability in biochar properties between locations, species and divisions

A multivariate resemblance matrix was produced to visualise multivariate properties of seaweed biochar from different locations, species and divisions. This resemblance matrix was visualised using a Canonical Analysis of Principal Coordinates (CAP) ordination with a vector overlay of Spearman rank correlations (limited to variables with a length of at least 0.5). The analysis included yield, C, H, O, N, S, P, K and pH as variables for each sample. The HHV and C/N data were excluded from the analysis as these are auto-correlated with the elemental profiles, while BET and CEC were excluded as they were only measured on individual samples for each species as described in the methods section. The data were 4^th^ root transformed to standardise the measurements and a similarity matrix was constructed from the transformed data using Primer 6, version 6.1.14 (Primer-E Ltd.).Two-tailed *t*-tests were also performed to test differences between red and brown seaweeds with respect to yield, elemental profile (C, H, O, N, S, P and K concentrations), pH, and CEC. The *t*-tests used mean values for each species to allow a specific contrast between red and brown seaweed groups.

## Author Contributions

D.R. conducted the analyses and wrote the manuscript; R.D.N. and N.A.P. obtained funding and assisted with experimental design; S.D. and M.I.B. obtained algal samples and prepared biochar. All authors reviewed the manuscript.

## Supplementary Material

Supplementary Information Supplementary Information

## Figures and Tables

**Table 1:** Yield and elemental composition of seaweed biochar. NQ – not quantified. C_Org_ is the % of total C content that is organic. All data are mean values ± S.E. and are calculated based on analysis of three independent biochar samples from each sample origin for each species. South Sulawesi and Java (Indonesia)

Species	Origin	Yield (%)	C (%)	H (%)	O (%)	N (%)	S (%)	HHV (MJ kg^−1^)	P (g kg^−1^)	K (g kg^−1^)	C/N
*Gracilaria*	*South Sulawesi*	59.8 ± 1.2	30.9 ± 0.1	2.2 ± 0.1	16.5 ± 0.4	2.8 ± 0.02	4.4 ± 0.1	16.1 ± 0.1	1.35 ± 0.05	51.2 ± 2.1	11 ± 0.1
	*Java*	61.8 ± 0.4	24.5 ± 0.01	1.5 ± 0.1	19.8 ± 0.2	1.3 ± 0.01	2.7 ± 0.5	11.1 ± 0.4	1.28 ± 0.20	116 ± 5	19 ± 0.1
*Eucheuma*	*South Sulawesi*	61.7 ± 2.1	25.6 ± 0.01	1.8 ± 0.04	24.9 ± 0.2	0.8 ± 0.03	9.3 ± 0.2	17.2 ± 0.1	1.78 ± 0.11	119 ± 3	31 ± 0.9
	*Java*	57.2 ± 0.3	23.7 ± 0.4	1.2 ± 0.03	20.6 ± 0.3	0.7 ± 0.01	7.0 ± 0.8	14.6 ± 0.6	0.91 ± 0.02	163 ± 4	33 ± 1.0
*Kappaphycus*	*South Sulawesi*	59.2 ± 5.3	31.3 ± 0.1	2.1 ± 0.03	23.8 ± 0.4	0.7 ± 0.01	6.8 ± 0.1	17.8 ± 0.1	0.51 ± 0.03	61.7 ± 2.3	46 ± 0.8
	*Kiribati*	54.1 ± 0.8	22.2 ± 0.2	1.1 ± 0.05	15.6 ± 0.2	0.3 ± 0.03	5.5 ± 0.1	13.0 ± 0.2	0.50 ± 0.01	158 ± 21	74 ± 7.3
*Saccharina*	*China*	49.7 ± 2.0	28.0 ± 0.2	1.9 ± 0.1	16.4 ± 0.2	2.2 ± 0.02	1.0 ± 0.01	11.4 ± 0.1	4.69 ± 0.20	8.9 ± 0.4	13 ± 0.2
	*Korea*	45.3 ± 0.2	35.0 ± 0.1	2.4 ± 0.1	18.4 ± 0.4	2.4 ± 0.01	1.6 ± 0.1	14.8 ± 0.2	6.60 ± 0.29	51.9 ± 1.3	15 ± 0.1
*Sargassum*	*China*	61.9 ± 1.6	28.9 ± 0.04	2.1 ± 0.01	18.2 ± 0.1	1.1 ± 0.01	2.8 ± 0.1	13.5 ± 0.1	1.60 ± 0.10	27.8 ± 0.8	27 ± 0.2
	*Indonesia*	49.0 ± 0.5	29.1 ± 0.1	2.0 ± 0.04	15.3 ± 0.2	1.0 ± 0.02	0.9 ± 0.04	11.8 ± 0.1	1.30 ± 0.03	31.1 ± 1.0	29 ± 0.5
*Undaria*	*China*	62.4 ± 0.3	34.8 ± 0.03	2.8 ± 0.03	15.6 ± 0.1	2.4 ± 0.1	0.8 ± 0.05	14.7 ± 0.1	3.91 ± 0.13	5.1 ± 0.4	15 ± 0.3
	*Korea*	60.3 ± 0.4	27.3 ± 0.4	1.7 ± 0.03	14.1 ± 0.1	2.3 ± 0.04	0.6 ± 0.2	10.7 ± 0.1	6.23 ± 0.40	9.0 ± 0.9	12 ± 0.2
Green macroalgae^2^	NA	50–73	10–35	0.8–1.5	NQ	1.1–3.3	NQ	NQ	1.7–5.5	NQ	8.5–12.2
Manure^12^	NA	33–57	27–52	NQ	NQ	NQ	NQ	NQ	0.7–4.4	1.0–3.6	NQ
Waste paper^12^	NA	37	56	NQ	NQ	NQ	NQ	NQ	0.1	0.1	NQ
Sawdust^12^	NA	28	76	NQ	NQ	NQ	NQ	NQ	0.1	1.2	NQ
Ligno-cellulosic^2^,^12^,^13^	NA	28–32	29–74	1.0–3.2	6.6–20.9	0.03–2.8	NQ	16.4–35.3	0.1–0.6	1.7–5.2	NQ
Microalgae^11^	NA	54–63	NQ	NQ	NQ	2.5–3.5	0.4	NQ	10	14–20	5.8–6.5

**Table 2:** BET surface area and pH of biochars produced from seaweeds. NQ – not applicable (not quantified by that study). The pH data are mean values ± S.E. and are calculated based on analysis of three independent biochar samples from each sample origin for each species. South Sulawesi and Java (Indonesia)

Species	Origin	BET (m^2^ g^−1^)	pH (CaCl_2_)
*Gracilaria*	*South Sulawesi*	2.02	7.6 ± 0.2
	*Java*	3.55	8.1 ± 0.1
*Eucheuma*	*South Sulawesi*	30.03	8.2 ± 0.2
	*Java*	34.82	8.6 ± 0.1
*Kappaphycus*	*South Sulawesi*	2.24	8.8 ± 0.1
	*Kiribati*	2.84	9.0 ± 0.1
*Saccharina*	*China*	1.29	11.0 ± 0.3
	*Korea*	8.48	11.2 ± 0.1
*Sargassum*	*China*	7.46	10.8 ± 0.1
	*Indonesia*	2.51	10.1 ± 0.2
*Undaria*	*China*	1.33	9.9 ± 0.1
	*Korea*	8.87	10.9 ± 0.1
Macroalgae (saltwater)^2^	NA	1.15–1.81	6.1–10
Macroalgae (freshwater)^3^	NA	8.29	7.8
Microalgae (saltwater)^11^	NA	10.7–20.7	7.2–7.9
Bagasse^10^	NA	259–452	NQ
Bagasse (activated)^10^	NA	441–570	NQ

**Table 3:** Exchangeable cations [cmol(+) kg^−1^] of seaweed biochar. NQ – not quantified. South Sulawesi and Java (Indonesia)

Species	Origin	Al	Ca	K	Mg	Na
*Gracilaria*	*South Sulawesi*	<0.1	22	280	13	100
	*Java*	<0.1	38	270	18	140
*Eucheuma*	*South Sulawesi*	<0.1	22	330	78	340
	*Java*	<0.1	19	390	100	330
*Kappaphycus*	*South Sulawesi*	<0.1	56	210	60	310
	*Kiribati*	<0.1	12	26	50	760
*Saccharina*	*China*	<0.1	4	320	26	200
	*Korea*	<0.1	12	120	52	430
*Sargassum*	*China*	<0.1	56	270	61	220
	*Indonesia*	<0.1	51	370	79	230
*Undaria*	*China*	<0.1	19	13	45	620
	*Korea*	<0.1	94	420	59	260
Ligno-cellulosic^3^	NA	NQ	21.8	12	NQ	1.09
Microalgae^11^	NA	NQ	9.4–18.3	12.2–13.9	6.0–10.7	36.6–58.8
