# Supplementary material for: Biochar from commercially cultivated seaweed for soil amelioration
Source: Sci Rep. 2015 Apr 9;5:9665. doi: 10.1038/srep09665 (PMC4391317; doi:10.1038/srep09665)

SUPPLEMENTARY INFORMATION

Title Biochar from commercially cultivated seaweeds for soil amelioration

Authors David Roberts[1*], Nicholas Paul[1], Symon Dworjanyn[2], Michael Bird[3], Rocky de Nys[1]

[1]MACRO – the Centre for Macroalgal Resources and Biotechnology, College of Marine and Environmental Sciences, James Cook University, Townsville Australia 4811

[2]National Marine Science Centre, Southern Cross University, Coffs Harbour Australia 2450

[3]Centre for Tropical Environmental and Sustainability Sciences, College of Science, Technology and Engineering, James Cook University, Cairns Australia 4870

***Corresponding:** david.roberts1@jcu.edu.au, (+617) 4781 3463.

Figure S1 Schematic of the muffle furnace retort used to produce the seaweed biochar. The main body of the retort has been made transparent to show the internal mechanism. The retort consists of a cylindrical stainless steel body. A lid screws onto the cylindrical body and a coiled tube passes through the lid to deliver N_2 into the apparatus. A ceramic mesh bag containing seaweed is held in place by the coiled N_2 delivery tube and N_2 gas is pumped into the apparatus from an attached N_2 gas cylinder. The entire apparatus is then inserted into a standard muffle furnace and heated to the desired temperature under continued N_2 flow at 4L min^{-1}. Gases produced during pyrolysis of the seaweed are vented through an exhaust vent in the lid of the cylindrical body. A hose is attached to the exhaust vent and directed to a water bath to trap gases and prevent reflux into the pyrolysis chamber.

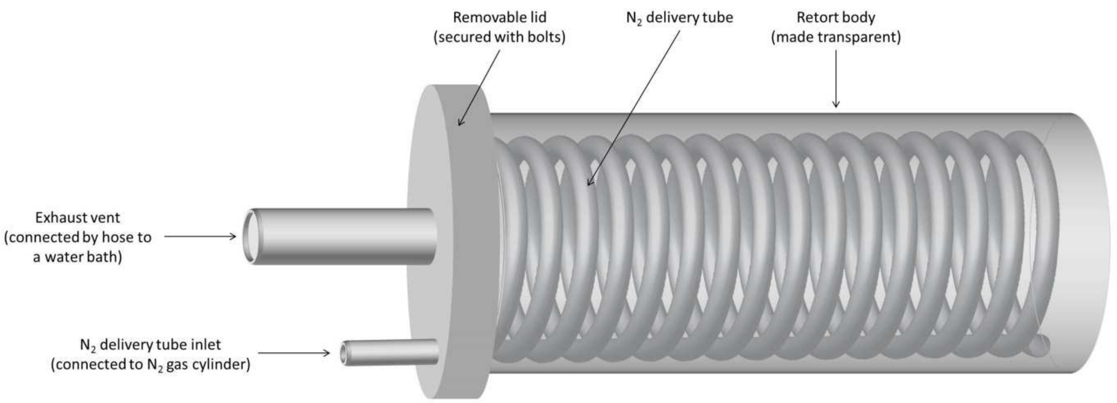

Supplement: Supplementary Information [file srep09665-s1.pdf]
